# Intercessory Rote Prayer, Life Longevity and the Mortality of Roman Catholic Bishops: An Exploratory Study

**DOI:** 10.1007/s10943-021-01214-9

**Published:** 2021-03-15

**Authors:** Natalia Banasik-Jemielniak, Dariusz Jemielniak, Wojciech Pędzich

**Affiliations:** 1 Institute of Psychology, Maria Grzegorzewska University, Warsaw, Poland; 2 Management in Networked and Digital Societies (MINDS) Department, Kozminski University, Warsaw, Poland

**Keywords:** Intercessory prayer, Faith healing, WikiData, Occupational longevity

## Abstract

Based on a computational analysis of a large dataset, this study explores if there is a significant longevity effect of intercessory prayer for a named individual’s well-being, if he receives a very high number of prayers per annum for an extended period. We relied on an observational cohort study, based on data from 1988 to 2018, including 857 Roman Catholic bishops, 500 Catholic priests, and 3038 male academics from six countries. We measured the covariance of the mean length of life, controlled for nationality. It was found that there is a main effect for occupation *F*(2, 4391) = 4.07, *p* = 0.017, *η*_*p*_^2^ = 0.002, with pairwise comparisons indicating significant differences between the mean life duration of bishops (*M* = 30,489) and of priests (*M* = 29,894), but none between the academic teachers (*M* = 30,147) and either of the other groups. A comparison analysis between bishops from the largest and the smallest dioceses showed no significant difference *t*(67.31) = 1.61, *p* = 0.11. The first analysis proved that bishops live longer than priests, but due to a marginal effect size this result should be treated with caution. No difference was found between the mean length of life of bishops from the largest and the smallest dioceses.

## Introduction

Intercessory prayer (praying on behalf of others) is sometimes perceived as a way of assuming social responsibility (Kormina & Luehrmann, 2018), as well as a dynamic ritual that makes social change possible (Shearer, 2015).

Even though assuming criteria of efficiency for prayer may be perceived as misleading, since prayer may be an activity with immaterial goals, “many Christians think that the success of an intercessory prayer is to be judged by the consequences that follow. Thus, they may ask if it leads to physical health. Did it restore the petitioner's psychic balance?” (Comstock, 1987, p. 693). Also, analyzing practical efficacy of prayer is common (Albanese, 1999; Ly et al., 2018).

Prayer is a practice with positive effects on the health of the praying (Hamilton et al., 2019; May et al., 2019). About 84% of the world population is religiously affiliated, and faith has a large influence on health-related decisions (Karam et al., 2015). A large number of people use personal prayer or spiritual healing as adjuncts to traditional care (Oman, 2018; Rao et al., 2015; Salsman et al., 2015). Such practices have proven to bring health benefits—for example in reducing depression and anxiety (Anderson & Nunnelley, 2016; Gonçalves et al., 2015) or overall psychological health of patients (Johnson, 2018; Naimi et al., 2018). Priests report effects of private prayers on their life satisfaction, higher than resulting from participation in public prayer (Büssing et al., 2016).

Distant healing or intercessory prayer was also reported to have positive effects in some trials (Astin et al., 2000; Sicher et al., 1998). Although criticized, there has been research suggesting that supplementary, blinded, remote, intercessory prayer may possibly produce a measurable improvement in the medical outcomes of coronary care unit patients (Byrd, 1988; Harris et al., 1999). Intercessory prayer is also sometimes associated, for example, with shorter stay in hospital and shorter duration of fever in patients with a bloodstream infection (Leibovici, 2001) as well as with reducing disruptive behavior of patients with dementia (Struve et al., 2016).

Other analyses insist that there is little empirical basis for assertions that such prayers can be associated with beneficial health outcomes (Sloan & Bagiella, 2002) and point out that non-contact healing studies are prone to a large degree of bias and errors (Roe et al., 2015). It is also clear that clinical efficacy of distant healing is not consistently observable (Radin et al., 2015). In fact, there is also research showing that intercessory prayer may worsen the patients’ condition, if they are aware of being prayed for (Benson et al., 2006), although its research design was criticized (Krucoff et al., 2006). Reliance on faith healing is also linked with higher mortality in children (Asser & Swan, 1998) and adults (Simpson, 1989). Still, the vast majority of patients accept offers of prayer on their behalf and find it helpful (McMillan & Taylor, 2018).

As Benor’s (2001) comprehensive review shows, there are lots of studies of medical effects of prayer and distant healing. Even though only about 25% of them follow a rigorous research design, three quarters of the rigorous ones reveal statistically significant results suggesting a healing effect of prayer. Levin (2020) believes that this finding has led to Benor’s systematic review being largely ignored in medical academia. It is worth noting that population studies of health and religion are particularly prone to reviewer and editor bias, coming both from skeptical and faith positions, and many academic journals avoid the topic if the results are not following the dominant theory (Levin, 2020). This may be one of the reasons why Byrd’s study (1988), relying on a double-blind, randomized controlled trial of distant intercessory prayer for coronary care patients, showing the efficacy of prayer, received such ardent critique, even though it was later replicated with statistically significant results, involving healers from different religious backgrounds (for a detailed analysis of Byrd’s study and the following discussion, see Levin, 2020:85–89, for methodological issues associated with intercessory prayer research see Aguiar et al., 2017).

Nevertheless, all contemporary studies of intercessory prayer rely on relatively small numbers of people praying for the well-being of a given patient or group of patients. Therefore, it is unclear if any effects of the prayer may be more noticeable when the number of the praying individuals, and the number of prayers in total, are significantly increased. Also, the studies so far have focused predominantly on people who already are struggling with a medical condition. The effects of being regularly prayed for while being in good shape have been rarely analyzed. Notably though, one of the early studies in statistical inquiries in 1872 did address the efficacy of prayer on the example of the British Royal Family as a whole (Galton, 2012). It did not focus on the effect of prayers targeting named individuals though.

In order to study the longevity effect of a large number of prayers for the well-being of specific, named individuals, we have decided to analyze the age at death for 857 bishops from 6 large Roman Catholic countries (the US, France, Italy, Poland, Brazil, and Mexico) over the last 30 years. This population is particularly interesting for our purpose, as Roman Catholic Church relies on Roman Missal (Vatican, 2010), a liturgical book, to structure the celebration of the Holy Mass. Although there have been several editions of this book over the last 50 years, they all include a part in which the congregation prays to the Lord, to guard the Pope and the local bishop, as well as reminds God: “Therefore, Lord, remember now all for whom we offer this sacrifice: especially your servant N. our Pope, N. our Bishop,* and the whole Order of Bishops” (where the asterisk is to be substituted with the name of the local bishop). During every single mass a local bishop receives a massive number of prayers meant for him specifically and individually, accompanied by a more general prayer for the well-being of all bishops as a group. Therefore, Catholic bishops are an ideal study group for our research, as there is a strong empirical support, based in a highly regulated liturgy, that they are prayed for on a regular basis by a large number of people.

Mass attendance in the chosen Catholic countries is relatively high. For instance, according to the most current statistics from Polish Roman Catholic Church, 36.7% of Catholics regularly participated in Sunday mass in 2016 (Konferencja Episkopatu, 2018). Given that this number is 9.8–12 million people according to different estimates (Konferencja Episkopatu, 2018; “Praktyki niedzielne Polaków,” 2017; Statistics Poland, Bureau, 2017), with an average of one attendance per week, and with the number of bishops in Poland around 150 (Konferencja Episkopatu, 2018), the total number of individual prayers received by one Polish bishop per annum exceeds 3 million. This number is similarly large for other studied countries (Cheney, 2018). With the exception of France, considered a secular country, where we estimate the number of prayers per bishop to amount to 330 thousand annually (“Liste des évêques, archevêques et Cardinaux,” 2018; Sawe, 2018), bishops from all other countries receive more than a million prayers per annum: in Italy slightly over 1 million (“I cattolici tra presenza nel sociale e nuove domande alla politica,” 2017), in the US about 2 million (“Bishops and Dioceses,” 2018, “Catholics by State,” 2014), in Brazil about 3.8 million (“Organização da Igreja no Brasil,” 2018; Rapoza, 2016), and in Mexico as many as 12 million (Cable, 2017; “Directorio Circunscripciones Eclesiásticas en la República Mexicana,” 2018). It should be noted that since the number of active mass participants has been systematically decreasing in all countries in time over the last century, the number of prayers received by the studied deceased population in the past may have been even higher.

If intercessory rote prayers and sacrifices for a specific named individual have an effect on longevity depending on the number of people praying, Roman Catholic bishops should live significantly longer, due to millions of prayers for their well-being and protection received by each of them every year. To analyze this phenomenon we chose two reference groups for comparison: other priests who did not become bishops and academic teachers.

Additionally, if the effectiveness of prayer grows proportionally to the number of the praying, bishops from larger dioceses should experience better longevity than their colleagues from the smaller ones. To address this possibility, we ran an additional analysis, comparing only bishops from the largest and the smallest dioceses in terms of Catholic population from all studied countries.

It should be noted that our study is hampered by incomplete data in terms of baseline health and socioeconomic status of the studied individuals, as well as other complex cultural factors, typical for studies of this size. Also, we focus on a very specific kind of intercessory prayer: the one highly regulated in the protocol of Roman Catholic mass, and not being said spontaneously by an individual, with unspecified intentionality and engagement. However, the sheer number of prayers per a named individual per annum make it an unparalleled outlier: it is safe to assume that no other group of individuals receives such a large number of prayers, and no other kind of prayers comes close in terms of quantity by several orders of magnitude. Thus, we believe that focusing on rote, unspontaneous prayers that are said by a large number of individuals in intention of a prayed-for person is useful. Levin’s meticulous analysis (2020: p. 96) shows that there are many population studies of health-effects of religion:

- Heart disease morbidity and mortality: 64 studies, 47 (73.4%) with positive findings.
- Hypertension and cerebrovascular disease: 87 studies, 55 (63.2%) with positive findings.
- Cancer morbidity and mortality: 84 studies, 64 (76.2%) with positive findings.
- All-causes mortality: 116 studies, 92 (79.3%) with positive findings.
- Self-rated health: 70 studies, 44 (62.9%) with positive findings.
- Pain and somatic symptoms: 118 studies, 50 (42.4%) with positive findings.
- Physical disability: 64 studies, 30 (46.9%) with positive findings.
- Depression: 459 studies, 317 (69.1%) with positive findings.
- Anxiety: 314 studies, 170 (54.1%) with positive findings.

However, to our knowledge there are no studies focusing on intercessory rote prayer for named individuals, in particular focusing on a very large number of prayers. Moreover, our approach, relying only on a large dataset of secondary data, is free of what many studies of intercessory prayer are prone to: the experimenter bias (Schlitz et al., 2006).

## Methods

We used data about the lifespan of all people in the chosen populations in six countries, who died over the last 30 years that was available on Wikidata.org. Wikidata is a volunteer-driven, collaboratively-developed (Jemielniak, 2016), multilingual secondary database (presenting not only statements, but also their sources as well as connections to other databases, e.g., identifiers in external sources) which provides structured data, readable by both humans and machines. While reliability of health information on Wikipedia and Wikidata is occasionally questioned, research shows that it is actually quite high (Matheson & Matheson-Monnet, 2017; Shafee et al., 2017). Additionally, it should be especially reliable and non-controversial for simple facts, such as the date of birth and the date of death (Smith, 2020).

In Wikidata, data is presented as items, each identified by a Q-number, of which there were nearly 50 million in August 2018. Each data item is presented with a set of statements, providing the detailed characteristics of the entry; statements consist of a property and a value or values corresponding to this property. Data in individual categories was extracted with the use of Wikidata Query service which allows to specify properties and their values to be included in the dataset, either through building a query with a user interface or writing a SPARQL query. Retrieved information included the date of birth and death, profession, and citizenship.

Our dataset consists of 857 catholic bishops, 500 catholic priests who were not bishops, and 3038 male academic teachers. The collection comprises all the individuals whose details were available in Wikidata with all the necessary properties present—date of birth, date of death, nationality, gender (in the case of academic teachers), and occupation. Bishops were manually excluded from the priest samples, as some individuals had both Wikidata properties populated with values, and academic teacher samples had all the priests and bishops removed. Similarly, bishops who held positions outside their country of birth as indicated in Wikidata were excluded from the sample. The subjects were listed as American, French, Italian, Polish, Brazilian, and Mexican nationals. All of them have died in the last 30 years and all of them were male.

The rationale behind choosing the professional groups for comparison was to compare a group of individuals who received an extensive number of intercessory prayers on a regular basis (bishops), a group that shared a lot of lifestyle choices with bishops, but received a vastly smaller number of intercessory prayers (priests, who are not mentioned in Roman missal and thus are not prayed for by default by any mass attendant in a diocese), and a third professional control group: male academics, who, like priests and bishops, have higher education, and share some social status similarity, as well as semi-structured workday scheme with bishops. The choice of encyclopedic male academics (typically, professors) was made also because we were looking for a profession outside of any religious affiliation, and yet combining relatively high social recognition, loosely regulated working hours, typical lack of hands-on supervision of the work process. Due to significant other differences in lifestyle and behavior this comparison should be treated with caution, however given the vast amount of intercessory prayers received by bishops, even if marginally effective, their influence should be significant and ostensible.

The SPSS software, version 25, was used to analyze the data. In order to compare the mean length of life among male bishops (*n* = 857), priests (*n* = 500) and academic teachers (*n* = 3038), we used a one-way analysis of covariance (ANCOVA). The independent variable, occupation, had three categories: bishops, priests, and academic teachers. The dependent variable was the length of life in number of days, and the covariate was the nationality. The assumptions for ANCOVA were met. Sidak correction for multiple comparisons was used to compare the adjusted means (controlled for the covariate) among the three occupation groups.

In the next step, we extracted the data about bishops that came from the largest and the smallest dioceses in terms of the number of the faithful belonging to the Catholic community. We later compared the length of life between the two groups of bishops—those coming from numerous religious communities and those coming from the small ones. In order to do that, we used the Student’s *t* test. Only bishops who had worked 10 years or longer in the largest or the smallest dioceses were included into the large/small religious community group, respectively. The length of life computed in days was taken to the final analysis for 126 bishops from the largest dioceses and for 47 bishops from the smallest dioceses. The smallest dioceses were the ones comprising of up to 700,000 members of the Catholic community. As the largest ones, we identified dioceses which gathered at least 1,000,000 members. Table 1 shows the contrast of fundamental demographic data within the three researched groups and general country population.

**Table 1:** Contrast of fundamental demographic data within the three researched groups and general country population in 2018

	Bishops		Priests		Academic teachers		General population	
	Gender *n* [%]	Education *n* [%]	Gender *n* [%]	Education *n* [%]	Gender *n* [%]	Education *n* [%]	Gender *n* [%]	Education *n* [%]
USA	Male 234 [100]	Tertiary 234 [100]	Male 109 [100]	Tertiary 109 [100]	Male 2142 [100]	Tertiary 2142 [100]	Male 181.6 mln [51.2] Female 173.1 mln [48.8]	Below secondary 16 mln [9.4] secondary 73.9 mln [43.4] tertiary 80.7 [47.4]
France	Male 108 [100]	Tertiary 108 [100]	Male 158 [100]	Tertiary 158 [100]	Male 380 [100]	Tertiary 380 [100]	Male 33.3 mln [51.2] female 31.7 mln [48.8]	Below secondary 6.7 mln [20.6] secondary 13.9 mln [42.5] tertiary 12.1 mln [36.9]
Italy	Male 205 [100]	Tertiary 205 [100]	Male 158 [100]	Tertiary 158 [100]	Male 212 [100]	Tertiary 212 [100]	Male 30.6 mln [51.5] female 28.8 mln [48.5]	Below secondary 12.6 mln [38.3] secondary 14 mln [42.4] tertiary 6.4 mln [19.3]
Poland	Male 48 [100]	Tertiary 48 [100]	Male 50 [100]	Tertiary 50 [100]	Male 184 [100]	Tertiary 184 [100]	Male 19.7 mln [51.5] female 18.5 mln [48.5]	Below secondary 16 mln [7.6] secondary 13.3 mln [61.5] tertiary 6.7 mln [30.9]
Brazil	Male 197 [100]	Tertiary 197 [100]	Male 30 [100]	Tertiary 30 [100]	Male 55 [100]	Tertiary 55 [100]	Male 107.8 mln [51.5] female 101.5 mln [48.5]	Below secondary 52.7 mln [47] secondary 38.8 mln [34.6] tertiary 20.6 mln [18.4]
Mexico	Male 65 [100]	Tertiary 65 [100]	Male 16 [100]	Tertiary 16 [100]	Male 66 [100]	Tertiary 66 [100]	Male 66.6 mln [51.5] female 62.8 mln [48.5]	Below secondary 37.4 mln [60.9] secondary 13 mln [21.2] tertiary 11.1 mln [18]

OECD, Adult education level, 2020, https://data.oecd.org/eduatt/adult-education-level.htm; United Nations, World Population Prospects 2019, https://population.un.org/wpp/DataQuery/

## Results

An ANCOVA [between-subjects factor: occupation (bishop, priest, academic teacher); covariate: nationality] revealed a main effect for occupation, F(2, 4391) = 4.07, *p* = 0.017, *η*_*p*_^2^ = 0.002. The pairwise comparisons are presented in Table 1. Contrast of average lifespans in days within the three researched groups with standard deviations and general country population in 2018 is presented in Table 2.

**Table 2:** Adjusted means for dependent variable (length of life in days) for Catholic priests, bishops, and academic teachers

Occupation	Mean	Std. Error	95% confidence interval	
			Lower bound	Upper bound
*Dependent variable: life length (days)*				
Priests	29,894,279	172,442	29,556,205	30,232,353
Bishops	30,489,014	138,900	30,216,699	30,761,328
Academic teachers	30,147,128	71,423	30,007,104	30,287,152

**Fig. 1:**
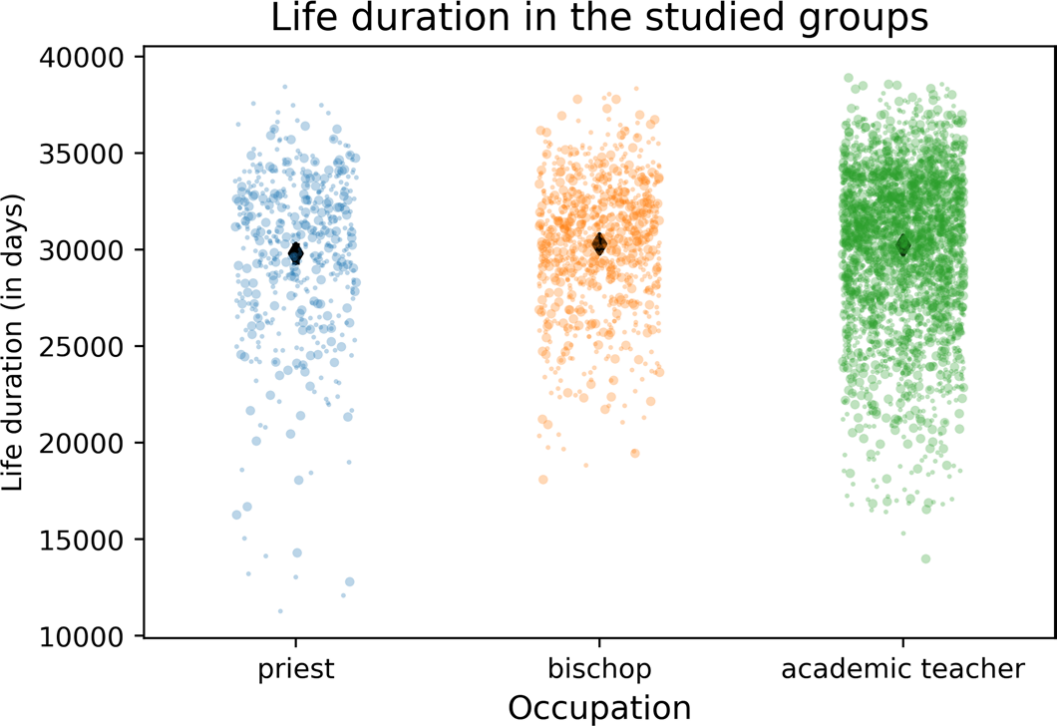
Life duration in the studied groups by occupation, each dot represents one individual, the diamond signifies the average longevity in days

**Fig. 2:**
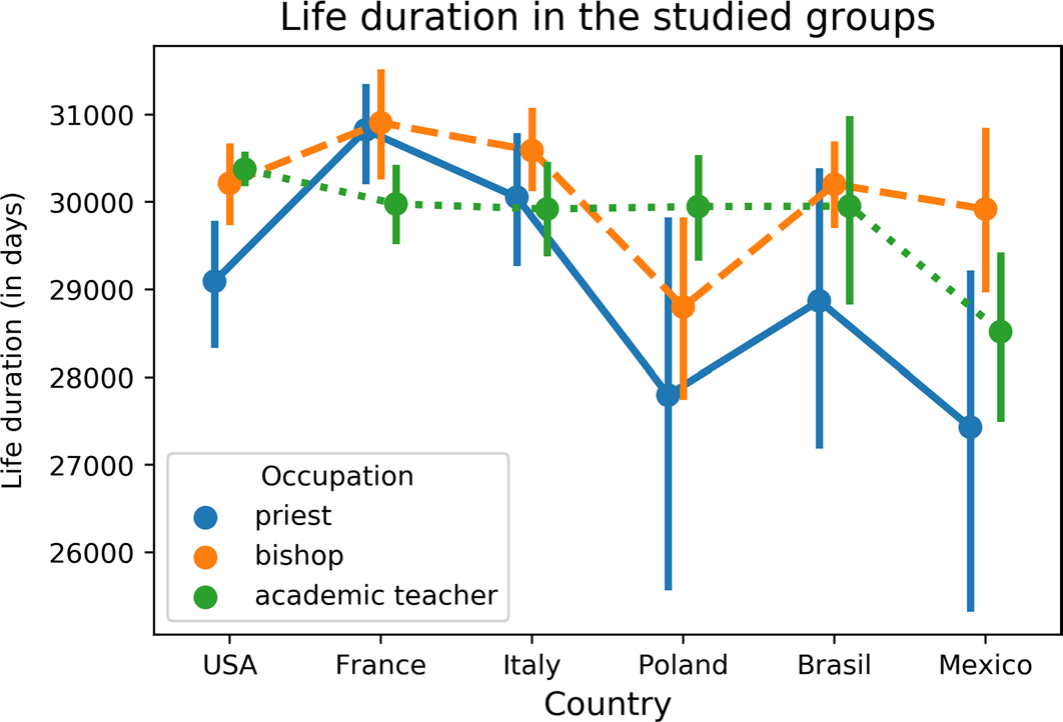
Average longevity in the study groups by country and occupation, whiskers are 95% confidence intervals

The *t* test analysis comparing the life length of bishops from large and small dioceses (hence, those receiving a higher and a lower number of prayers, respectively) showed that there is no significant difference between the group of bishops from the largest (*M* = 30,926.13 SD = 2869.485), and the bishops from the smallest (*M* = 29,959.23, SD = 3734.55) dioceses *t*(67.31) = 1.61, *p* = 0.11. The confidence intervals of the bishops sample and the priests sample overlap slightly (Table 3, Figs. 1, 2).

**Table 3:** Contrast of average lifespans in days within the three researched groups with standard deviations and general country population in 2018 (United Nations Development Programme, 2018)

	Bishops			Priests			Academic teachers			General population
	*M*	SD	*n*	*M*	SD	*n*	*M*	SD	*n*	*M*
USA	30,215	3400	234	29,093	3725	109	30,375	3821	2142	29,037
France	30,906	3064	108	30,824	3550	158	29,978	4118	380	30,206
Italy	30,588	3215	205	30,054	4401	158	29,920	3772	212	30,389
Poland	28,799	3691	48	27,700	5957	50	29,948	3960	184	28,416
Brazil	30,203	3275	197	28,870	4385	30	29,952	4033	55	27,649
Mexico	29,918	3675	65	28,336	3234	16	28,517	4035	66	28,234

## Discussions and Limitations

The ANCOVA was significant, which means that there is a main effect for occupation. Pairwise comparisons of adjusted means with Sidak correction showed that Catholic bishops live significantly longer than Catholic priests. No difference was found between the length of life of male academic teachers and bishops or between priests and male academic teachers. However, it is important to note that the effect size was marginal. The partial squared eta indicates that the occupation can only account for 0.2% of the variance in the length of life.

A further analysis with Student’s *t* test showed no significant difference between the means of life length of bishops coming from the largest and from the smallest dioceses.

Hence, although it seems that bishops do live longer than priests (but not longer than academic teachers), we can also conclude that the number of prayers one receives does not influence the duration of life. If this was the case, we would expect a significant difference between the two groups in the *t* test analysis.

Our study relies on analyzing the age at death for Roman Catholic bishops, and compares their longevity with notable priests and male academic teachers from the same countries. Since we are relying only on data of people who were notable enough to be reflected on Wikidata, which in turn relies to a large degree on data imported from Wikipedia, our sample is not representative of all priests or all scholars. However, we consider it a strength of the paper, as encyclopedic notability may be a factor nullifying some of the income and social class differentiation.

Our study is the first study of intercessory prayer that attempts to account for a very large number of prayers per capita, received over a period of many years, and meant for a named individual. A possible limitation to our study is that we do not account for the intensity or intentionality of the prayer. After all, a large number of mass participants may not necessarily put a lot of dedication or emotions into the particular request for a given bishop’s well-being, and the bishop is also a barely known stranger to the most of the praying. Additionally, the rote prayer, mechanically repeated as a part of mass procedure is definitely significantly different from a personal, intimate, and ardent prayer one would employ if they actually wanted to ask for someone’s health (Dossey, 2008).

Also, our study is limited only to Roman Catholic church. Another limitation is lack of spatial proximity to the bishop, as in some studies of faith healing the proximity plays an important role (Astin et al., 2000; Roe et al., 2015). Additionally, the Roman Catholic prayer for the local bishop, while clearly individually aimed, is not very specific in terms of the requested result. The prayer for well-being and protection is not explicitly phrased as a request for longer life or health. Nevertheless, we believe that it is quite likely that the vast majority of both the praying, and the prayed for would possibly agree that health and long life are the desirable outcomes of the prayer. Finally, one of the weaknesses of the study is the sample size. Even though Wikidata has millions of records, in the future there is likely going to be significantly more bishops, notable priests, and scholars covered, as the saturation is still increasing rapidly.

Our results may be explained in a number of ways. Firstly, there might be factors that were not included in the analysis that influence the duration of life in general, which possibly outscore or interact with the effect of millions of prayers. A factor that tends to be of the same value for priests and bishops while being different for academic teachers is the family. Catholic clergy are obliged to live in solitude, do not marry nor have children. It has been shown that supportive personal relationships such as with marital partners or children do influence subjective life expectancy (Ross & Mirowsky, 2002), which has been proven to predict the actual mortality (van Solinge & Henkens, 2018). We might hypothesize that even if the prayers do prolong the subjects’ lives, the lack of close family relations might significantly decrease the life expectancy for clergymen while increasing it for academic teachers, which would explain why there is a difference between bishops and priests, but not for either of the two groups and academic teachers.

Secondly, bishops have a significantly higher material status than regular priests. Even though precise salary comparisons are not always possible, due to their discretionary nature and cross-country differences, it is undisputed that bishops are at the top of the church pay scale. Additionally, bishops typically have most of the regular costs of living, such as accommodation, meals, and full health benefits, or even a chauffeur, fully covered (Forgey, 2014; Witkowska, 2011). Thus, their compensation is largely available for strictly personal expenses, and as such quite high. Regular priests do not receive such perks, and their salaries are also lower, often significantly below the country’s median for people with higher education (Hoge, 2002; Proeschold-Bell et al., 2013). Given that the income-mortality gradient is much higher at low to moderate income levels (Backlund et al., 1996; Marmot, 2002; Mortensen et al., 2016), the wealth factor could contribute to bishops’ mortality advantage over the regular priests. On a similar note, bishops come from families with higher social origins than local clergy, and can be considered a social elite (Reese, 1983), which may definitely have an effect on their comparatively better longevity.

Thirdly, we cannot exclude the possibility that in the group of bishops, the individuals are, to a certain degree, pre-selected for longer life. That is, since the bishops are appointed after having met various qualifications having served as priests (Reese, 1983), and in our sample the vast majority became bishops not earlier than in the last three decades of their lives, whereas priests usually enter the profession at a young age, some priests pass away before reaching the age when bishops are appointed.

Finally, we do realize that the main effect which was found should be interpreted with caution since the effect size was marginal, and the occupation can only account for a very small percentage of the variance.

The lack of difference in means of length of life between bishops from the largest and the smallest dioceses means we found no effect of the number of received prayers on life duration. Hence, the hypothesis of the prayer effect on length of life was not confirmed.

As Jeff Levin (2020) notes, studies of the effectiveness of prayer on health and longevity are highly ideologized, and may be weaponized by secular skeptics and devout Christians alike. Thus, we would like to emphasize once more that our study’s focus was only on highly regulated and formalized intercessory rote prayer, being a part of an organized mass. This is the only kind of prayer that we are aware of that provides millions of prayers for a named individual per annum and as such deserves closer investigation. However, our study by design does not reflect on any prayers being said specifically by an individual on their own with a clear and personal intention of improving someone’s well-being, or on prayers being said by groups of individuals with a clear intention and an emotional investment. Future research of intercessory prayer may answer the questions of the effect of prayer intensity and wish commitment, as well as variations between different religions. Given the growing trust of academics in Wikipedia (Jemielniak & Aibar, 2016), as well as a vast increase in Wikidata coverage in general and medical content in particular (Shafee et al., 2017), these, and other occupational longevity studies are likely to appear in the near future. Additionally, an analysis of work-related stress influence on longevity of bishops in comparison to priests could shed new light on the discussed problem.
